# Supplementary material for: Integrative Utilization of Transcriptomics and Metabolomics Sheds Light on Disparate Growth Performance of Whiteleg Shrimp, Litopenaeus vannamei
Source: Int J Mol Sci. 2025 Mar 28;26(7):3133. doi: 10.3390/ijms26073133 (PMC11988672; doi:10.3390/ijms26073133)
Supplement: Supplementary file 1 [file ijms-26-03133-s001.zip › Table S1.pdf]

Table S1 Primers used in the present study for qPCR validation.

No.	Genes ID	Primer sequence (5’-3’)
1	LOC113815133	GATACCTGTGCGCTTGTGGTTG
		ACTTGAGGGTGAGGATACCACG
2	LOC113816317	ATATAACCCGGCCATCCCTCAG
		TTGCGGAGGGTCTCAATCTG
3	LOC113812620	TTTAAGGAGTTCACCCGCACG
		ATATCATCTCCCTGCCGTCCAC
4	LOC113816686	AACGGCTTCCTCCCTGACTTC
		TTCATCGGATGGTAGAAGAGCTGG
5	LOC113815517	GAGGACACTGAGGAGAACAAACTG
		CGTCAATGGCTTCACTCTTGC
6	LOC113814652	GGAGGAGATTAGGAAATGGGAGTTG
		CTCGCGGACCATACGCATTTG
7	LOC113803929	AACGAGTGTTCAACGGACAACG
		TCTTGGCACGAGAGTTTGTCC
8	LOC113815271	CCCAGCATTCTTTGGGACATTTC
		TCACGTAGATACAGACACTGTGC
9	LOC113828893	GTCAAGGTCATCAAGCAGCATC
		ACCTTATTGGGCACCTTCTCC
10	LOC113820678	TTCGGAAGCACTTTGAGACAGC
		AGGCCATGACTTGGTTCTCTTG
11	*GAPDH*	CCCTTCATCACGCTGGACTAC
		AACACACCAGTGGACTCAACGA
