# Supplementary material for: Integrative Utilization of Transcriptomics and Metabolomics Sheds Light on Disparate Growth Performance of Whiteleg Shrimp, Litopenaeus vannamei
Source: Int J Mol Sci. 2025 Mar 28;26(7):3133. doi: 10.3390/ijms26073133 (PMC11988672; doi:10.3390/ijms26073133)
Supplement: Supplementary file 1 [file Figure S1.pdf › Figure S1.pdf]

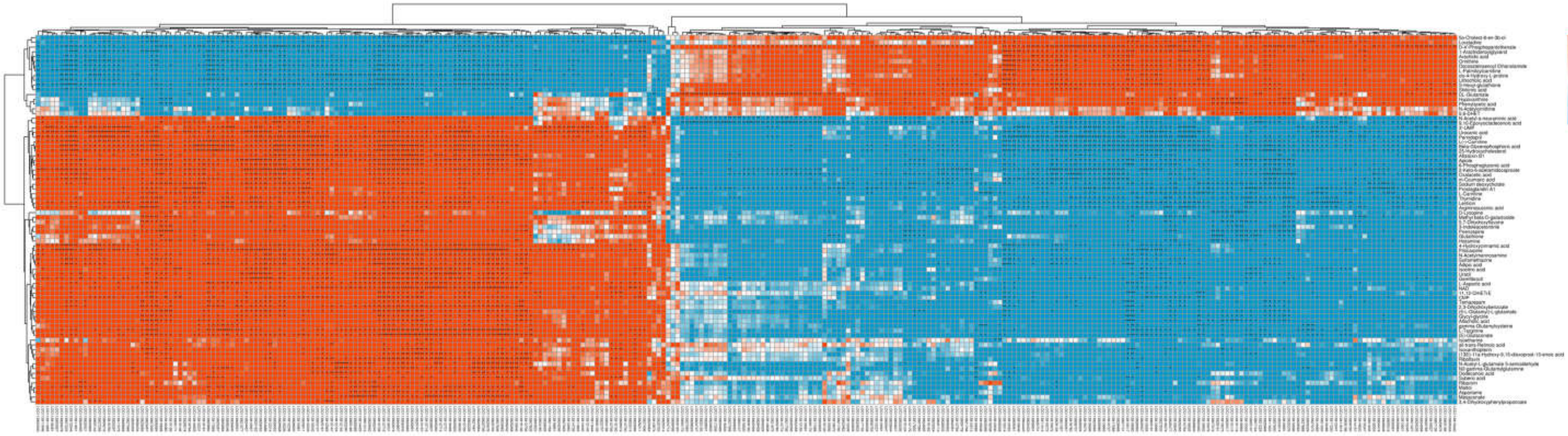

**Supplementary Figure S1.** Correlation analysis between the transcriptome and metabolome of *Litopenaeus vannamei*. “*” denotes $p < 0.05$, “**” denotes $p < 0.01$, and “***” denotes $p < 0.001$.
